# Aptamers for Targeted Drug Delivery

**DOI:** 10.3390/ph3061761

**Published:** 2010-05-27

**Authors:** Partha Ray, Rebekah R. White

**Affiliations:** Department of Surgery, Duke University Medical Center, DUMC Box 103035, Durham, NC 27710, USA

**Keywords:** Systemic Evolution of Ligands by Exponential Enrichment (SELEX), aptamers, targeted drug delivery

## Abstract

Aptamers are a class of therapeutic oligonucleotides that form specific three-dimensional structures that are dictated by their sequences. They are typically generated by an iterative screening process of complex nucleic acid libraries employing a process termed Systemic Evolution of Ligands by Exponential Enrichment (SELEX). SELEX has traditionally been performed using purified proteins, and cell surface receptors may be challenging to purify in their properly folded and modified conformations. Therefore, relatively few aptamers have been generated that bind cell surface receptors. However, improvements in recombinant fusion protein technology have increased the availability of receptor extracellular domains as purified protein targets, and the development of cell-based selection techniques has allowed selection against surface proteins in their native configuration on the cell surface. With cell-based selection, a specific protein target is not always chosen, but selection is performed against a target cell type with the goal of letting the aptamer choose the target. Several studies have demonstrated that aptamers that bind cell surface receptors may have functions other than just blocking receptor-ligand interactions. All cell surface proteins cycle intracellularly to some extent, and many surface receptors are actively internalized in response to ligand binding. Therefore, aptamers that bind cell surface receptors have been exploited for the delivery of a variety of cargoes into cells. This review focuses on recent progress and current challenges in the field of aptamer-mediated delivery.

## 1. Introduction

**Figure 1:**
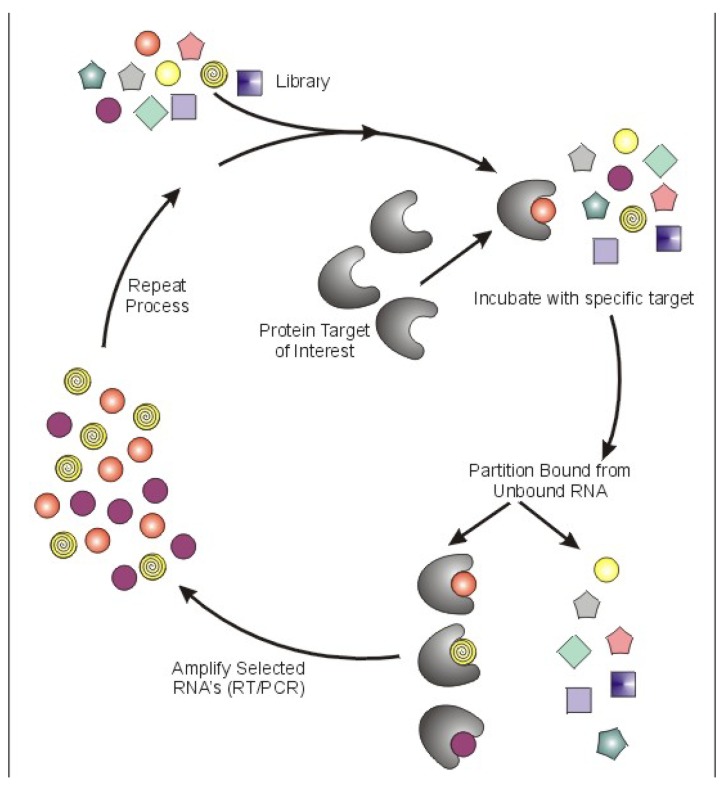
Schematic of Systematic Evolution of Ligands by Exponential Enrichment (SELEX).

Aptamers are a class of therapeutic oligonucleotides that form specific three-dimensional structures that are dictated by their sequences. In contrast to antisense oligonucleotides and small interfering RNAs (siRNAs) that inhibit translation of proteins by Watson-Crick base-pairing to their respective messenger RNAs, aptamers bind to existing proteins (and, less commonly, non-protein targets) with high affinity and specificity, analogous to monoclonal antibodies. Aptamers are typically generated by an iterative screening process of complex nucleic acid libraries (>10^14 ^shapes per library) employing a process termed Systematic Evolution of Ligands by Exponential Enrichment (SELEX) [1,2]. The SELEX process consists of iterative rounds of affinity purification and amplification; over successive rounds, the pool becomes enriched for ligands that bind the target protein with high affinity and specificity (Figure 1). Several unique properties of aptamers make them attractive tools for use in a wide array of molecular biology applications and, moreover, as potential pharmaceutical agents. First, most aptamers bind to targets with high affinity, demonstrating typical dissociation constants in the pico- to nanomolar range. Binding sites for aptamers include clefts and grooves of target molecules (including enzymes) resulting in antagonistic activity very similar to many currently available pharmaceutical agents. Second, aptamers are structurally stable across a wide range of temperature and storage conditions, maintaining the ability to form their unique tertiary structures. Third, aptamers can be chemically synthesized, in contrast to the expensive and work-intensive biological systems needed to produce monoclonal antibodies. 

RNA and DNA aptamers both have theoretical advantages and proponents, but aptamers of comparable affinity and specificity can be generated from RNA or DNA. Since nuclease resistance is critical for aptamer stability in biological fluids, RNA libraries employed in SELEX are front-loaded with 2'-modified nucleotides, most commonly 2'-fluoro- or 2'-*O*-methyl pyrimidines [3]. Probably the biggest current advantage of DNA over RNA aptamers is the significantly lower cost of chemical synthesis for unmodified DNA oligonucleotides. However, RNA is preferred by many groups due to the theoretically higher affinity of RNA aptamers for their target proteins as well as the greater plasma stability of modified RNA than unmodified DNA. Stimulation of the immune system via Toll-like Receptors by double-stranded regions within RNA aptamers is a valid concern [4], but modified (“artificial”) nucleotides do not appear to be potent stimulators of this innate immune response [5].

Most SELEX libraries have random regions ranging from 20 to 60 nucleotides (nts), flanked by constant regions for amplification and transcription, and therefore total lengths ranging from 70 to greater than 100 nts. Sufficient quantities of any length aptamer can be generated by *in vitro* transcription for testing *in vitro* and in certain small scale animal models. However, chemical synthesis is necessary for larger scale applications. Since the efficiency of chemical synthesis decreases with oligonucleotide length, it is often necessary to “minimize” or “truncate” an aptamer prior to study *in vivo*. Although the technology continues to improve, aptamers longer than 60 nts are not currently amenable to cost-effective chemical synthesis, and the shorter, the better. Due to the relatively small size (8 kDa to 15 kDa) of truncated aptamers, their circulating half-lives are limited not by plasma stability but by renal clearance, which can be improved by conjugation to high molecular weight groups such as polyethylene glycol (PEG) [6].

**Table 1:** Cell surface protein aptamers and their applications.

Receptor Name	RNA/DNA	Selection Technique	Delivery Application
Tenascin-C (TN-C)	RNA	Purified TN-C	*In vivo* tumor imaging [25]
Nucleolin	DNA	Not applicable	Photodynamic therapy (PDT) [26], tumor imaging [27]
Prostate Specific Membrane Antigen (PSMA)	RNA	Purified extracellular domain of PSMA	siRNA delivery [28,29,30], cytotoxin delivery [31], Chemotherapeutic drug delivery and cellular imaging [32,33,34]
gp120	RNA	Purified recombinant gp120	siRNA delivery [35]
Transferrin receptor (TfR)	RNA/DNA	Purified extracellular domain of mouse TfR	Protein targeting to lysosome [13]
Mucin-1 (MUC-1)	DNA	Recombinant peptides	Photodynamic therapy (PDT) [36], Radionuclide delivery [37]
Protein tyrosine kinase-7 (PTK7)	DNA	Cell SELEX using T-cell acute lymphoblastic leukemia (ALL) cell line	Chemotherapeutic drug delivery [38]
Immunoglobin heavy mu chain (IGHM)	DNA	Cell SELEX using Burkitt’s lymphoma cell line (Ramos)	Micelle nanoparticles for drug delivery [39]
Epidermal growth factor receptor (EGFR)	RNA	Purified extracellular domain of EGFR	Nanoparticle delivery [22]

Over the past two decades, this technology has enabled the generation of aptamers to a myriad of proteins including reverse transcriptases, proteases, cell adhesion molecules, infectious viral particles, and growth factors (reviewed in [7,8,9,10]). Although not a new concept, aptamers have only become realistic clinical agents as methods for their efficient synthesis have improved, similar to monoclonal antibodies 30 years ago. Intraocular delivery of a nuclease-resistant RNA aptamer against vascular endothelial growth factor (VEGF) modified with PEG (pegaptinib or Macugen®, Eyetech Pharmaceuticals) is now in clinical use for the wet form of age-related macular degeneration [11]. 

SELEX has traditionally been performed using purified proteins, and cell surface receptors may be challenging to purify in their properly folded and modified conformations. Therefore, relatively few aptamers have been generated that bind cell surface receptors. However, improvements in recombinant fusion protein technology have increased the availability of receptor extracellular domains as purified protein targets, and the development of cell-based selection techniques has allowed selection against surface proteins in their native configuration on the cell surface. With cell-based selection, a specific protein target is not always chosen, but selection may also be performed against a target cell type with the goal of letting the aptamer choose the target. The past decade has seen the generation of several aptamers that bind to cell surface receptors [12,13,14,15,16,17,18,19,20,21,22]. All cell surface proteins cycle intracellularly to some extent, and many surface receptors are actively internalized in response to ligand binding. Therefore, aptamers that bind cell surface receptors have been exploited for the delivery of a variety of cargoes into cells (see Table 1 as well as previous reviews [23,24]). This review focuses on recent progress and current challenges in the field of aptamer-mediated delivery. 

## 2. Tenascin-C

Tenascin-C (TN-C) is an extracellular matrix protein (ECM) that is implicated in the process of tissue remodeling. It is overexpressed in the tumor stroma where it is thought to enhance angiogenesis and invasion [40,41]. In one of the earliest examples of “blind” cell-based SELEX for target identification, Daniels *et al.* [42] performed selection against U251 glioblastoma cells. The resulting DNA aptamer was used for affinity purification of the target, which was identified by mass spectrometric analysis as TN-C. Although this study helped to validate TN-C as an aptamer target, this particular aptamer did not bind with high affinity to TN-C at physiologic temperatures. The same group therefore performed a selection against purified TN-C using a 2’fluoro-pyrimidine-modified RNA library [43]. A truncated version of the winning aptamer was further modified by replacing purines with 2’-OMe-modified purines and capping the 3’ end. A 5’-amine was incorporated that was used to conjugate the metal chelator MAG2. The MAG2-aptamer was subsequently radiolabeled with ^99m^Tc. In order to assess the tumor uptake and biodistribution property of the radiolabeled anti-TN-C aptamer it was injected intravenously into mice bearing gliobastoma (U251) and breast cancer (MDA-MB-435) tumor xenografts. Scintigraphic images of the tumors were taken 18 hours after injection and revealed that the aptamer was exclusively localized in tumors. This was achieved due to the combined effect of the efficient uptake of aptamer by the tumor and its rapid clearance from the blood [25]. This proof-of-principle study demonstrated that an anti-TN-C aptamer could be used to target tumors (glioma, breast, and colon) that express high level of TN-C and that aptamers, in general, may have pharmacologic properties that make them excellent agents for tumor-specific drug delivery. 

## 3. Prostate-Specific Membrane Antigen

Prostate-specific membrane antigen (PSMA) is a type II membrane-associated metallopeptidase that is overexpressed on the surface of prostate cancer cells. PSMA is also expressed in the vasculature of many other solid tumors [44]. Therefore, it qualifies as an important prostate cancer marker and an attractive anti-cancer therapeutic target. Lupold *et al.* [14] selected anti-PSMA aptamers by using the extracellular portion of purified recombinant PSMA and a 2’-fluoropyrimidine modified library. Two aptamers (A9 and A10) were selected that demonstrated specific binding to PSMA. This was evaluated by the ability of the aptamers to inhibit the enzymatic activity (*N*-acetylated-α-linked-acidic-dipeptidase) of PSMA. A truncated version of A10 was created that bound to a PSMA-positive (LNCaP) prostate cancer cell line. The binding was selective, because A10 did not bind the PSMA-negative PC3 prostate cancer cell line. 

Based on these findings and the previous knowledge that PSMA is internalized via clathrin-coated pits to endosome [45], three different groups tested the possibility of using the anti-PSMA aptamer for the purpose of siRNA delivery into cells. The idea was that the anti-PSMA aptamer would carry the attached siRNA to the cells that express PSMA, and the aptamer-siRNA bound to the PSMA protein would gain access to the cell via internalization. Next, the siRNA portion would undergo processing by the Dicer complex and feed into the RNA-Induced Silencing Complex (RISC)-mediated gene-silencing pathway. 

These groups utilized different strategies to accomplish this goal. Chu *et al.* [28] used a biotin-streptavidin bridge mediated conjugation method to assemble the anti-PSMA (A9) aptamer and the siRNA. The following chemical manipulations were performed to achieve it: (1) 3’ end of A9-aptamer was biotinylated, (2) 5’-biotin was conjugated to the sense strand of an anti-lamin A/C or GAPDH (Glyceraldehyde 3-phosphate dehydrogenase) siRNA, and (3) the biotinylated aptamer and the siRNA were incubated together with streptavidin in a 2:2:1 ratio to assemble the aptamer:siRNA:streptavidin complex. The knock-down efficiency of the aptamer:siRNA:streptavidin was comparable to the positive control where the siRNA was introduced into the cell by the use of lipid based transfection reagent. Additionally, unlike the lipid based transfection method, the aptamer:siRNA:streptavidin mediated gene knock-down was target cell specific as there were no gene knock-down in PSMA-negative PC3 cells. Importantly, the immune response that is often associated with siRNA delivery was not observed with this *in vitro* application. This was the first demonstration that aptamers could be used to deliver lethal siRNAs to targeted cancerous cells. 

McNamara *et al.* [29] also demonstrated this aptamer mediated siRNA delivery approach. However, instead of using the A9 aptamer, these authors used the anti-PSMA aptamer (A10) for this purpose. Moreover, instead of using the biotin-streptavidin bridge method, they used a “RNA-only” aptamer-siRNA chimera approach to link the aptamer and the siRNA. Namely, the 3’ end of the A10 aptamer was extended to include the upper strand of the siRNA and the lower anti-sense strand was later annealed to it to form the aptamer-siRNA chimera. For the targeted gene knock-down experiment, the authors selected siRNA against two survival genes PLK1 (Polo-like kinase 1) and BCL2. Knock-down of targeted genes and consequently reduced cell proliferation and apoptosis were observed in PSMA expressing LNCaP cells. No such effects were observed in the control PSMA-negative PC3 cells. In order to test the *in vivo* efficacy of this delivery approach, the authors directly injected the chimera into LNCaP tumors that were established in mice. As a control, the same application was performed on mice bearing PC3 (PSMA-negative) tumors. Mice bearing the LNCaP tumors showed reduction in the tumor volume due to the chimera injection (but not the injection control chimeras), whereas no effect was observed in the PC3 tumors. This study clearly demonstrated the specificity and efficacy of the aptamer mediated siRNA delivery approach. In a recent study done by the same group [46], the chimera was truncated and modified to enhance its bioavailability and therapeutic efficacy. When administered systemically into mice bearing PSMA-expressing tumor xenografts, the aptamer-chimera resulted in the shrinkage of tumors. 

In a subsequent study by Wullner *et al.* [30], the authors used the anti-PSMA aptamer to deliver Eukaryotic Elongation Factor 2 (EEF2) siRNA to PSMA-positive prostate cancer cells. Bivalent PSMA aptamers were used for this purpose. The authors demonstrated that, compared to the monovalent anti-PSMA-siRNA chimera, the gene knock down potency of the bivalent aptamer-construct was superior. 

In parallel, a series of other studies demonstrated that PSMA aptamers could be exploited in a variety of ways to deliver cargo into cells. Gelonin is a ribosomal toxin that can inhibit the process of protein synthesis and is cytotoxic. However, it is membrane impermeable and needs an usher for its cellular entry. In one of the first examples of aptamer-mediated delivery, Chu *et al.* [31] realized that the anti-PSMA aptamer could be employed to deliver this toxic payload into prostate cancer cells that express PSMA. Gelonin was directly conjugated to A9, and the aptamer-toxin construct was incubated with LNCaP cells. *In situ* immunofluorescence microscopic analysis and cytotoxic assay demonstrated that the conjugate was internalized into the cells and resulted in targeted cell killing. 

Since direct conjugation limits the amount of cargo that each aptamer can deliver, other strategies have incorporated aptamers as targeting moieties for a variety of functional polymers and nanoparticles, each of which can carry many molecules. For example, tumor resistance to cytotoxic chemotherapeutic agents is due in part to insufficient delivery to and uptake by cancer cells. Biodegradable nanoparticles (NP) derived from poly(D,L-lactic-*co*-glycolic acid) PLGA were used to solve this targeting problem [34]. Cisplatin was converted to its pro-drug, Pt(IV) compound, by introducing two alkyl chains. This increased the hydrophobicity of the compound and eased the process of its packaging within the hydrophobic core of the NP. Polyethylene glycol (PEG) was used as a copolymer during the nanoprecipitation step to synthesize the PLGA-PEG nanoparticle. It is known that PEG-nanoparticles increase drug bioavailability due to decreased systemic clearance. For prostate cancer cell targeting, PLGA-PEG-NP surface was decorated with PSMA aptamer A10. The NP underwent endocytosis when incubated with LNCaP cells, and the alkylated pro-drug was converted to cisplatin by the cytosolic reduction process. *In vitro* cytotoxicity assays done with LNCaP cells demonstrated that the A10 conjugated Pt(IV) prodrug-PLGA-PEG-nanoparticle was more potent than either free cisplatin or nontargeted (aptamer non-conjugated) nanoparticles. Farokhzad *et al.* [33] had previously used the same A10-PLGA-PEG-NP approach to deliver Docetaxel (Dtxl). Nanoparticle containing Dtxl was injected directly into LNCaP tumors. Dramatic tumor reduction and prolonged survival were observed in the experimental animals. 

Aptamers are adept at multi-tasking. Bagalkot *et al.* [32] demonstrated that aptamers could simultaneously function as drug delivering and imaging agent. These authors used a quantum dot (QD)-aptamer-doxorubicin (Dox) conjugated nanoparticle for this purpose. The A10 aptamer was first conjugated to the surface of a fluorescent quantum dot (QD). As demonstrated by the authors [32], this provided the QD with its prostate cancer cell targeting potential. Next, the QD-aptamer conjugate was incubated with Dox to form the QD-aptamer-Dox nanoparticle. Both Dox and QD are fluorescent molecules. However, due to their proximity in the QD-aptamer-Dox nanoparticle, they quench each other’s fluorescence by a bi-fluorescence resonance energy transfer (FRET) mechanism. Thus, the QD-aptamer-Dox nanoparticle is non-fluorescent. However, the internalization of the QD-aptamer-Dox nanoparticle via PSMA-mediated endocytosis in prostate cancer cells causes the release of Dox from the QD-aptamer-Dox nanoparticles, that results in the recovery of fluorescence by both Dox and QD. Cell proliferation assays were also performed on LNCaP cells, demonstrating that QD-aptamer-Dox was as cytotoxic as free Dox. Moreover, the toxicity of QD-aptamer-Dox was minimal on PSMA negative PC3 cells demonstrating the PSMA mediated specific targeting potential of QD-aptamer-Dox. Kim *et al.* [47] recently used anti-PSMA aptamer-conjugated polyethylenimine and PEG polyplexes to co-deliver small hairpin RNA (shRNA) against anti-apoptotic gene Bcl-xL and chemotherapeutic drug (Dox) to selectively and potently kill LNCaP cells *in vitro*. These creative examples of combinatorial approaches demonstrate that aptamer-conjugated polymers and nanoparticles may be powerful platforms for targeted drug delivery and imaging with minimal toxic side effects. 

## 4. gp120

HIV-1 (Human Immunodeficiency Virus), the causative agent of AIDS (Acquired Immuno Deficiency Syndrome) is an enveloped retrovirus [48,49] that employs the envelope glycoprotein (Env) gp160 to bind and infect T-cells. gp120 binds the T-cell receptor CD4 [50] and one of the chemokine co-receptors, either CCR5 or CXCR4 [51], and mediates fusion between the virus particles and the host T-cells [52]. Viral entry therefore can be blocked by targeting either the viral gene product gp120 and gp41 or the host T-cell receptor CD4 and the co-receptors, CCR5 or CXCR4. Accordingly, Khati *et al.* [12] selected 2’-fluoropyrimidine modified RNA aptamers against the gp120 of the R5 strain, HIV-1_Ba-L_ that blocked the gp120-CCR5 interaction [53] and neutralized the infectivity of a broad range of HIV-1 primary isolates [54]. Zhou *et al.* [35] of the Rossi group realized that gp120 is internalized by host cells that have been infected by HIV-1 and can be targeted to deliver anti-viral (tat/rev) siRNA using the anti-gp120 aptamer. The anti-gp120 aptamer-siRNA chimera demonstrated superior HIV-1 inhibitory effect in comparison to the anti-gp120 aptamer alone, thus suggesting cooperativity of action between the siRNA and the aptamer portion of the chimera. In order to test the efficacy of siRNA processing the authors compared chimeras containing either a 27-mer or 21-mer duplex RNA. They found that the chimera with 27-mer duplex RNA demonstrated more efficient gene silencing than the corresponding 21-mer duplex containing chimeras. In a more recent study, the same group selected several new 2’-fluoropyrimidine modified RNA aptamers against the same gp120 target [55]. To one of their aptamers they introduced a 16-nt 3’ “sticky-bridge” that could be potentially used to link more than one type of siRNA to the same aptamer for delivery. This approach could be potentially used to simultaneously down-regulate multiple target genes and, in the case of HIV-1 infection, prevent the virus from developing resistance to a single siRNA. Since the expression of gp120 on the cell surface is restricted to HIV-1 infected cells, siRNA delivery would be constrained to only the infected cells, which would minimize the potential off-target effects of the siRNAs. 

## 5. Nucleolin

Nucleolin, first described by Orrick *et al.* [56] is a predominantly nuclear and cytoplasmic phosphoprotein that has been implicated to play key roles in diverse biological processes [57]. In addition to its nuclear and cytoplasmic localization, several groups have reported the presence of nucleolin at the cell surface, and nucleolin is believed to shuttle to and from the cell surface via mechanisms that have not been elucidated. The expression of nucleolin on the cell surface is correlated with cell proliferation [58]. Consequently, the nucleolin levels are higher in tumors and other actively dividing cells [59]. Moreover, cell surface nucleolin functions as a receptor to various growth factors like midkine and pleiotrophin that can transform cells [60]. Therefore, the functional blockage of the cell surface nucleolin represents a potential target for the development of anti-cancer therapeutics. 

A 26-nucleotide guanosine-rich (G-rich) DNA sequence (AS1411) was discovered serendipitously by Bates *et al.* to have anti-proliferative activity and subsequently found to bind nucleolin. This aptamer was therefore not “selected” in the way that most of the other aptamers described in this review were. It was further established that AS1411 inhibited the pro-survival NF-κB signaling pathway [61] and thus blocked DNA-replication and induced cell cycle arrest and apoptosis. Another mechanism of action that contributes to the anti-cancer effects of AS1411 was described in a separate study conducted by Soundararajan *et al.* Anti-nucleolin aptamer was found to inhibit the binding of nucleolin to Bcl-2 mRNA. This resulted in the destabilization of the mRNA with a consequent decrease in the level of anti-apoptotic Bcl-2 protein in the breast cancer cells [62]. Studies conducted by Ireson *et al.* [63] in nude mice bearing tumor xenografts derived from breast and lung cancer cells demonstrated anti-tumor effects of AS1411 *in vivo*. Currently, the anti-nucleolin aptamer AS1411 is in phase II clinical trails for acute myeloid leukemia and renal cell carcinoma. 

In addition to the direct anti-cancer effects of AS1411, the fact that nucleolin shuttles between the cell surface, cytoplasm, and nucleus in rapidly dividing cells means that, in principle, nucleolin can be used to deliver cargo into cancer cells. A precedent for this has already been set. A 34-amino acid peptide (F3) discovered by Christian *et al.* [59] using a phage-display technique was shown to recognize nucleolin expressed on the cell surface of endothelial cells present in angiogenic vessels. This tumor homing peptide was also internalized and transported to the nucleus. Banking on this initial observation, Drecoll *et al.* used radio-labeled F3-peptide to deliver α-particle emitting ^213^Bi isotope into the nucleus of tumor cells in the mouse intraperitonial xenografts model [64]. Reduction in tumor volume and increase in the survival time of mice due to this application were promising results.

However, the unusually high serum stability and low immunogenicity of the anti-nucleloin aptamer AS1411 [63] make it a potentially better choice over peptides for the purpose of tumor targeting. AS1411 has recently been used as an imaging probe for cancer cells. This function of AS1411 was reported by Hwang *et al.* who conjugated it with a multimodal nanoparticle (MFR-AS1411) and monitored uptake into C6-rat glioma cells by using fluorescence confocal microscopy. For the *in vivo* tracking of MFR-AS1411, it was injected systemically into nude mice bearing C6 tumor xenografts subjected to both whole body scintigraphic and Magnetic Resonance (MR) imaging techniques. Using these *in vivo* and *in vitro* methods, these authors have demonstrated that the anti-nucleolin aptamer can target nanoparticles to cancer cells expressing nucleolin on their cell surface and can potentially be used as a non-invasive imaging tool for the diagnosis of cancer [27]. Recently, Shieh *et al.* [26] used AS1411 as a carrier to deliver a photosensitizing agent (5,10,15,20-tetrakis(1-methylpyridinium-4-yl)porphyrin or TMPyP4) into MCF7 (breast cancer) cells. TMPyP4 was non-covalently complexed with the AS1411 aptamer for this purpose. *In vitro* studies demonstrated that the aptamer-TMPyP4 complex was readily taken up by MCF7 cells expressing the nucleolin and were severely damaged in response to photodynamic therapy (PDT) as compared to the control normal epithelial cells. Given the observation that AS1411 is internalized at much lower concentrations than those necessary for anti-proliferative effects [62], AS1411 is a particularly promising agent for these and other targeted delivery approaches. 

## 6. Transferrin receptor (TfR)

TfR is a ubiquitously-expressed membrane bound protein that is involved in the process of iron uptake into cells and thus maintains cellular iron homeostasis. After transferrin-Fe^+3^ binds TfR, the transferrin-Fe^+3^-TfR complex undergoes endocytosis and is transported to the endosomal compartment where the iron is released and the TfR-apotransferrin is recycled back to the plasma membrane [65,66]. To test the idea that anti-TfR aptamers can be used to carry cargoes into cells, Chen *et al.* [13] selected RNA and DNA aptamers using the purified extracellular domain of mouse TfR. The selected aptamers were biotinylated and subsequently linked to Cy5 fluorophore-labeled streptavidin. Using confocal microscopy, it was established that the dye-labeled aptamers were bound and internalized into mouse fibroblast cells. The aptamers were specific to mouse TfR as it failed to bind human 293T cells unless they were transfected with the mouse TfR encoding gene. As mentioned above, after endocytosis, the TfR-apotransferrin complex is recycled back to the cell membrane from the endosomal compartment. However, the fluoroscently-labled aptamers followed a different intracellular route. The labled aptamers reached the lysosome, as verified by their co-localization with a lysosome staining dye (dextran-Texas red). It should be noted that typically TfR-apotransferrin is not directed to the lysosome, but there are reports of it being rerouted to the lysosomal compartment under certain conditions [67]. Prompted by this finding, the authors next conjugated a lysosomal enzyme α-L-iduronidase to the DNA aptamer for the purpose of delivering it into mouse fibroblast cells that were deficient in the enzyme. It was found that the enzyme-aptamer conjugate could correct the accumulation of glycosaminoglycan (GAG), a substrate for the α-L-iduronidase enzyme, thus suggesting that the anti-TfR aptamer was able to deliver an enzymatically active protein to its destined organelle, the lysosome. This elegant method could be applied to correct various lysosomal storage diseases. Moreover, in principle, this technique can be adapted to target other enzymes into subcellular compartments using aptamers against receptors that are internalized by endocytosis. 

## 7. Mucin-1(MUC-1)

MUC-1 is a cell surface associated glycoprotein that is extensively modified by *O-*glycosylation. Aberrant and incomplete glycosylation of MUC-1 is often associated with various epithelial cancer cells (breast, ovary, colon, pancreas lungs and prostate). These abnormally glycosylated proteins, termed as glycoforms, represent a valuable class of tumor biomarkers because they are expressed only on cancer cells and are distinct from those expressed on normal cells [68]. Studies have shown that these under-glycosylated MUC-1 proteins are internalized via clathrin-mediated endocytosis into the lysosomal and Golgi-compartments [69]. Aptamers generated against the MUC-1 glycoforms have recently been used to deliver drugs into cancer cells. Ferreira *et al.* [36] selected three DNA aptamers that could selectively bind to human breast and pancreatic cancer cell lines expressing MUC-1. Using flow cytometric analysis, it was further established that the aptamers were internalized into these cell lines. Monodansylcadaverine, an inhibitor of receptor-mediated endosytosis, significantly inhibited the aptamer entry into the cells, thus suggesting the involvement of this pathway in aptamer internalization. Next, a photosensitizing agent chlorin *e*_6_ was coupled to the 5’ amino group of the MUC-1 aptamers and targeted to cancer cells expressing the aberrant MUC-1 glycoforms. These conjugated MUC-1 aptamers produced cytotoxic singlet oxygen species upon photodynamic therapy (PDT) and displayed greater than 500 times enhanced cellular toxicity as compared to when chlorin *e*_6_ was used as a free drug. Interestingly, normal human mammary cells that express the fully glycosylated MUC-1 were not affected by the conjugated MUC-1 aptamers upon PDT thus demonstrating remarkable cancer cell-targeting specificity of these aptamers [36]. Pieve *et al.* [37] from the same group recently conjugated MUC-1 aptamers with a chelating ligand mercapto-acetyl diglycine (MAG_2_) and subsequently radio-labeled them with the isotope ^99m^Tc. The ^99m^Tc-MAG_2_-conjugated aptamers were injected systemically into mice bearing MCF7 (breast cancer cell) xenografts, and the bio-distribution was studied. The aptamer-radionuclide was taken up by tumor cells suggesting these aptamers may also be useful for the diagnosis and staging of breast cancer. 

## 8. Protein tyrosine kinase 7 (PTK7)

PTK7 is a membrane bound receptor tyrosine kinase-like molecule. It is over-expressed in colon carcinomas and is also known as colon carcinoma kinase-4. Although it contains a catalytically inactive tyrosine kinase domain, it has been suggested to retain a role as a signal transducer in some tumors types [70]. A DNA aptamer that binds to PTK7 was developed by Shangguan *et al.* [71]. However, it should be noted that the selection was not intended to find an anti-PTK7 aptamer. The selection was performed with the intention to identify new cancer cell specific biomarkers using a cell-SELEX protocol. The authors used a T-cell acute lymphoblastic leukemia (ALL) cell line, CCRF-CEM, as the target for aptamer selection. Ramos (human Burkitt’s lymphoma) cells were used for counter selection to prevent the enrichment of DNA aptamers that could recognize common molecules present on the surface of both cell lines. A 41-nt aptamer sgc8c, that demonstrated remarkable specificity in terms of binding to the CCRF-CEM cells was further characterized [71]. Using protein purification methods, the target that bound to the aptamer was isolated and subsequently identified by mass-spectrometric analysis to be PTK7 [72]. In subsequent studies done by the same group and others, it has been demonstrated that the aptamer sgc8c is internalized into target cells [38,73,74]. In the study conducted by Tong *et al.* [73], the sgc8c aptamer was conjugated to the surface of a viral capsid protein (MS2) by using chemoselective oxidative coupling reaction to construct a biodegradable drug-delivery vehicle. The interior of the viral capsid was modified and linked to the AlexaFluor 488 maleimide in order to detect it in cell-binding assays. Flow-cytometric analysis revealed the binding of the aptamer-cojugated viral capsid to the targeted Jurkat T leukemia cells. Using confocal technique it was established that the capsids were internalized into the cells and co-localized with a lysosomal marker (Low Density Lipoprotein). As another confirmation of internalization, Kang *et al.* [75] recently used sgc8-conjugated liposome nanostructures as a platform to deliver low molecular weight dextran conjugated to FITC as a model drug into CEM-CCRF cells for confocal imaging. 

The sgc8c aptamer is therefore a promising candidate for targeted drug delivery. Huang *et al.* [38] covalently conjugated the anthracycline chemotherapeutic agent doxorubicin (Dox) to the sgc8c aptamer using an acid-labile hydrazone linkage. The idea was that, upon binding the receptor PTK7, the conjugate would be internalized and transported to the endosome. Under the influence of endosomal low pH, the acid labile arm that holds the Dox to the aptamer would be broken and would release the Dox to diffuse to the nucleus where it can intercalate with the chromosomal DNA, stop the replication process, and thus kill the targeted cancer cell. By using flow-cytometric and confocal microscopic techniques, it was demonstrated that sgc8c-Dox bound the CCRF-CEM cells and was internalized. Two hours after internalization of the sgc8c-Dox conjugate, it was found that autofluorescent Dox managed to escape the endosomal compartment and was evenly distributed through out the cell. Using a 3-(4,5)-dimethylthiahiazol-2-yl)-2,5-diphenyl-tetrazolium bromide (MTT) assay, the viability of the cells that were treated with sgc8c-Dox was measured. Interestingly, the IC_50_ of sgc8c-Dox was found to be same as free-Dox. 

More recently, Taghdisi *et al.* [76] used the sgc8 aptamer to deliver daunorubicin, another anthracycline chemotherapeutic agent, to PTK7 expressing acute lymphoblastic leukemia T cells (Molt-4). Flow cytometric analysis demonstrated that the sgc8-daunorubicin complex was internalized by the Molt-4 but not by the control U266, a PTK7 negative cell line. Consequently, the aptamer-drug complex was less toxic to U266 cells as compared to the daunorubicin alone. In yet another application, Kang *et al.* [75] recently used sgc8-conjugated liposome nanostructures as a platform to deliver small molecule drugs into CEM-CCRF cells. 

Anthracycline family-based chemotherapeutic drugs are membrane permeable and are randomly taken up by the cells through the process of passive diffusion. However, conjugating them with the sgc8c aptamer restricts their entry into cells that express PTK7. This “sieve” mechanism should curb the non-specific uptake of chemotherapeutic drugs and minimize the toxic effects of chemotherapeutic agents on normal cells.

## 9. Immunoglobin Heavy Mu Chain

Immunoglobin heavy mu chain (IGHM) is the large polypeptide subunit of the IgM antibody. IGHM expression level on premature B-cell correlates with the development of Burkitt’s lymphoma [77,78]. As described above for PTK7, selection of an aptamer against IGHM was not intentional but resulted from a cell-SELEX experiment done by Mallikaratchy *et al.* [79] with the objective to find an aptamer that could bind selectively to a Burkitt’s lymphoma cell line, Ramos. One of the resulting aptamers (TD05) that could recognize the target Ramos cell was used to identify its interacting protein partner. The aptamer was modified (post-selection) with photoactive uracil derivative (5-dUI) for this purpose. This facilitated the process of cross-linking the aptamer with its target protein on the cell membrane and simplified the protein purification step. Mass spectrophotometric analysis of the purified protein latter identified the target as IGHM. The aptamer, which had been identified during a selection performed at 4 °C, was not bound or internalized at 37 °C. As part of what could be considered an ingenious bioengineering solution, TD05 was linked with a PEG and lipid tail that helped it to be assembled into a micelle, which enhanced binding affinity to Ramos cells at 37 °C. Next, the aptamer-micelle was loaded with a dye that could fluoresce only when it is inside the cell. After incubating this dye-loaded aptamer-micelle with the cells, fluorescence microscopic images were taken, and it was established that the aptamer-micelle could fuse with the cell membrane and release its content into the cytoplasm of its targeted cell. Using a simplified flow chamber that mimics the physiological circulatory system, it was further demonstrated that the aptamer-micelle could target cells under dynamic fluid conditions [39]. This proof-of-concept study demonstrates that relatively low affinity aptamers can be tailored into aptamer-micelle nanostructures and can be used to deliver hydrophobic drugs into the targeted cells. 

## 10. Epidermal Growth Factor Receptor (EGFR)

EGFR is a transmembrane receptor tyrosine kinase and is considered the “prototype” for receptor-mediated endocytosis. Binding of EGFR to its cognate ligand causes receptor dimerization leading to autophosphorylation, internalization of the receptor, and activation of intra-cellular signal transduction pathways [80]. EGFR over-expression is associated with variety of cancers and linked to poor prognosis and decreased survival. Direct inhibition of EGFR by monoclonal antibodies (cetuximab) and small molecules (erlotinib) has been proven beneficial in some but not all tumor types. Additionally, since EGFR is internalized upon ligand-binding, it would seem to be an ideal target for aptamer-mediated delivery of cytotoxic drugs into the cancerous cells. Li *et al.* [22] recently selected a RNA aptamer against EGFR. Using flow cytometric and confocal microscopic techniques, it was established that the aptamer bound a human epithelial carcinoma cells line, A431, which expresses high levels of EGFR. Moreover, a novel RNase based assay was developed by these authors that in conjunction with the flow-cytometric analysis demonstrated the internalization of the aptamer in these cells. An interesting application of the aptamer as demonstrated by these authors is that gold nanoparticles (GNP) coated with the anti-EGFR aptamer were specifically targeted and internalized into high EGFR-expressing cell line A431 but not the low EGFR-expressing breast cancer cell line MDA-MB-435. Thus, this aptamer can in principle be used to deliver drugs or other cytotoxic cargo into cancer cells that express EGFR. The particular aptamer used in this study is made of unmodified RNA that might have *in vivo* stability issues; therefore, these authors have also generated a modified RNA aptamer against EGFR that is currently being tested (Li, unpublished observations).

## 11. Summary

Aptamers are nucleic acid ligands with several properties that make them attractive as pharmaceutical agents. Aptamers bind their targets with high affinity and specificity and are amenable to large-scale chemical synthesis. The versatility of the aptamer selection process has facilitated the generation of aptamers that bind a wide array of targets, including several cell surface receptors. Aptamers which bind cell surface receptors that are internalized have been exploited to deliver a variety of cargoes into cells. Aptamers therefore may be used to deliver molecules that are not otherwise taken up efficiently by cells (e.g., siRNAs) or to limit delivery of molecules that are efficiently taken up by cells (e.g., most chemotherapeutic agents) to cells that express aptamer targets.

The perfect target for aptamer-mediated delivery is one that is highly expressed on all target cells, is efficiently internalized, and is not expressed on the surface of non-target cells. Although a perfect target may not exist, several aptamers against excellent targets have been identified. Studies using these aptamers have provided “proof of concept” that aptamers can mediate cell type-specific delivery. Cargoes have included enzymes, toxins, chemotherapeutic agents, imaging agents, and siRNAs. Current challenges are both to optimize the cargo (what to attach and how to attach it) and to identify even better targets (and aptamers that bind them). With respect to the former, the fields of nanotechnology and RNA interference are rapidly maturing and will result in even more sophisticated aptamer constructs. With respect to the latter, further refinement of cell-based SELEX and *in vivo* SELEX techniques should facilitate the identification of additional cell type-specific targets and aptamers. 

Meanwhile, although we have an abundance of promising *in vitro* data, we have only a modest amount of *in vivo* animal data and—to date—no human data demonstrating that aptamer-mediated delivery is feasible. One factor that has potentially slowed the preclinical development of some aptamer therapeutics is the cost of synthesis, which may be prohibitive for aptamers longer than 40–50 nucleotides, particularly if the application requires repeated and/or systemic delivery. However, as methods for the chemical synthesis of olignonucleotides have improved, the yields and costs of synthesis have improved. The synthesis of oligonucleotides is also very “scalable”, as evidenced by the growing number of olignonucleotide therapeutics entering clinical trials. Furthermore, by using aptamers to deliver highly potent cargo, the amount of aptamer required (and therefore synthesis costs) may be significantly less than required for using aptamers as direct inhibitors. Therefore, we anticipate that aptamer-mediated delivery will prove to be feasible *in vivo* and that translation of this approach to human patients is a realistic goal for the near future. 
